# Trends in mitochondrial unfolded protein response research from 2004 to 2022: A bibliometric analysis

**DOI:** 10.3389/fcell.2023.1146963

**Published:** 2023-03-24

**Authors:** Zelin Ye, Ruoning Chai, Yujie Luan, Yihang Du, Wenjing Xue, Shuqing Shi, Huaqin Wu, Yi Wei, Limei Zhang, Yuanhui Hu

**Affiliations:** Department of Cardiology, Guang’anmen Hospital, China Academy of Chinese Medical Sciences, Beijing, China

**Keywords:** mitochondrial unfolded protein response, bibliometrics analysis, Citespace, UPR mt, VOSviewer, bibliometrix

## Abstract

The mitochondrial unfolded protein response (UPR^mt^) is a stress response pathway that regulates the expression of mitochondrial chaperones, proteases, and other proteins involved in protein folding and degradation, thereby ensuring proper mitochondrial function. In addition to this critical function, the UPR^mt^ also plays a role in other cellular processes such as mitochondrial biogenesis, energy metabolism, and cellular signaling. Moreover, the UPR^mt^ is strongly associated with various diseases. From 2004 to 2022, there has been a lot of interest in UPR^mt^. The present study aims to utilized bibliometric tools to assess the genesis, current areas of focus, and research trends pertaining to UPR^mt^, thereby highlighting avenues for future research. There were 442 papers discovered to be related to UPR^mt^, with the overall number of publications rising yearly. *International Journal of Molecular Sciences* was the most prominent journal in this field. 2421 authors from 1,402 institutions in 184 nations published studies on UPR^mt^. The United States was the most productive country (197 documents). The top three authors were Johan Auwerx, Cole M Haynes, and Dongryeol Ryu. The early focus of UPR^mt^ is “protein.” And then the UPR^mt^ research shifted from *Caenorhabditis elegans* back to mammals, and its close link to aging and various diseases. The top emerging research hotspots are neurodegenerative diseases and metabolic diseases. These findings provide the trends and frontiers in the field of UPR^mt^, and valuable information for clinicians and scientists to identify new perspectives with potential collaborators and cooperative countries.

## Introduction

Mitochondria are commonly known as cellular energy centers. They generate the majority of the cell’s energy by combining nutrient oxidation through the respiratory chain with ATP synthase to produce ATP. In addition to producing energy, mitochondria have other important roles, including cellular signal transmission, maintaining calcium homeostasis, and synthesizing numerous cofactors such as heme (Kořený et al., 2022), iron-sulfur clusters (Lill and Mühlenhoff, 2005), lipoic acid (Schonauer et al., 2009), and coenzyme Q (Bentinger et al., 2010), which are essential for a range of metabolic pathways in the cell. Furthermore, while mitochondria do not directly regulate cell differentiation or growth, their functions can have an impact on these processes. Therefore, maintaining mitochondrial protein homeostasis is critical for proper mitochondrial function.

Indeed, UPR^mt^ is a crucial mechanism that regulates mitochondrial protein homeostasis in response to various cellular stresses (Mahmud et al., 2020). The UPR^mt^ plays an essential role in maintaining mitochondrial function by inducing the expression of mitochondrial chaperones and proteases, which aid in the proper folding and management of misfolded proteins. Conditions that elicit the UPR^mt^ response include disruptions in oxidative phosphorylation (OXPHOS), excessive reactive oxygen species (ROS) production, impaired complex assembly, and accumulation of misfolded proteins. These conditions can result in mitochondrial dysfunction, leading to decreased ATP production and impaired cellular respiration, ultimately compromising cellular homeostasis and causing detrimental effects. The activation of the UPR^mt^ in response to stress is crucial to ensure the maintenance of cellular energy metabolism. Without this response, misfolded proteins would accumulate, leading to the formation of toxic aggregates, which can damage the organelle and the entire cell (Nargund et al., 2012a).

The discovery of UPR^mt^ in *Caenorhabditis elegans* and mammalian cells has led to significant progress in comprehending the response to mitochondrial stress. Recent findings indicate that UPR^mt^ is a multifaceted response that can be elicited by diverse mitochondrial stressors beyond protein misfolding. Moreover, it has become apparent that UPR^mt^ not only responds to mitochondrial stress but also participates in regulating an array of cellular processes, including metabolism (Lin and Haynes, 2016), inflammation (Zhu et al., 2023), and aging (Schulz and Haynes, 2015). To elucidate the regulatory mechanisms of UPR^mt^, researchers have identified various components necessary for its activation, including sensors of mitochondrial dysfunction, such as ATFS-1 (Nargund et al., 2012b) in *C. elegans* and HSPB1(Mule et al., 2021) in mammals, as well as regulators of mitochondrial-to-nuclear communication, such as the mitochondrial protease ClpP (Haynes et al., 2007) and the transcription factor CHOP(Horibe and Hoogenraad, 2007). Additionally, chromatin regulators, such as the histone deacetylase Sirtuin 1 (Brenmoehl and Hoeflich, 2013), have been shown to be involved in UPRmt activation. In conclusion, UPR^mt^ is a complex cellular response that can be activated by a spectrum of mitochondrial stressors and plays a pivotal role in regulating diverse cellular processes. Further exploration of the intricacies of UPR^mt^ regulation may provide valuable insights into the pathophysiology of mitochondrial dysfunction-related diseases and their treatment.

Bibliometrics is a research method used to analyze publications both qualitatively and quantitatively. This method allows researchers to gain immediate insight into the thematic evolution, primary study domains, and future research paths in a certain research field (Weingart, 2005). Bibliometrics is now frequently employed as an auxiliary research tool in a wide range of subjects. However, there are few bibliometric studies on UPR^mt^. In this study, we utilized bibliometric approaches to evaluate the research state, present research emphasis, and develop research trends in the field of UPR^mt^ during the 2004 to 2022, identifying potential avenues for future research.

## Materials and methods

### Data source and search strategy

**FIGURE 1:**
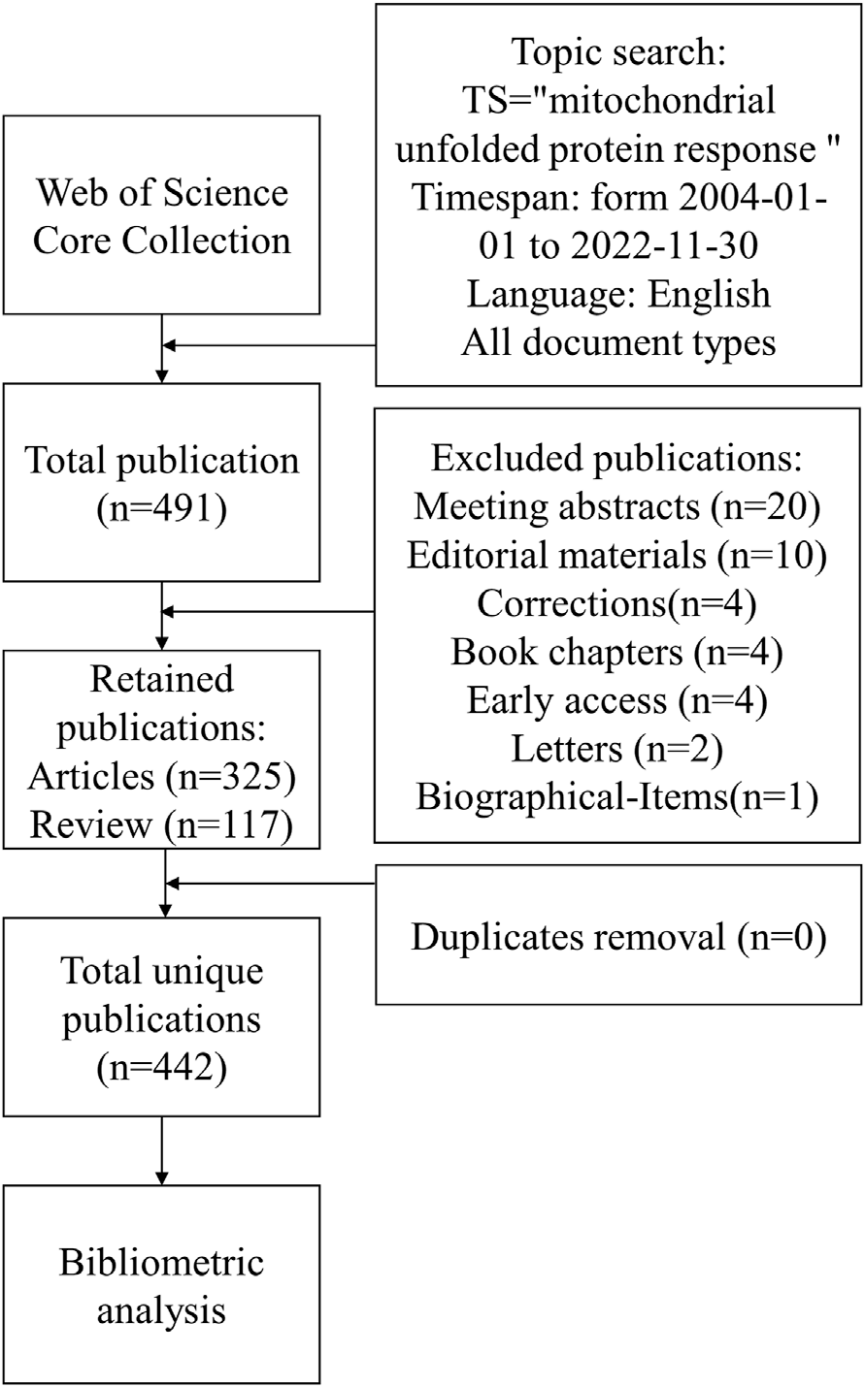
The flowchart illustrating the search formula and selection process in this study.

The data from Web of Science Core Collection (WoSCC) has the most complete data structure, including publication type (PT), author (AU), journal (SO), keyword (DE), abstract (AB), institution (CI) and reference (CR). Another reason it was selected as the data source is that its data is compatible with the visualization function of Citespace. At the same time, in order to ensure comprehensive and accurate retrieval of data, only the Science Citation Index Expanded (SCI-Expanded) and Social Sciences Citation Index (SSCI) were utilized. Based on the publication date of the first paper obtained during the preliminary search, the retrieval time frame was set from 2004 onwards. Figure 1 displays the search formula and inclusion criteria. The following were the inclusion criteria for the publication: 1) UPR^mt^ research; 2) the kind of publications comprised articles, reviews, and openly available data; 3) The publication was published in English. The following criteria were used to exclude the publication: 1) The articles did not deal with the study’s issue; 2) The publications were news, conference abstracts, or briefs. All the above operations will be completed within 1 day on 30 November 2022, and only the data downloaded on that day will be used.

### Eligibility criteria and data collection

Only articles and reviews were accepted as document types. Duplicate studies were manually removed. For bibliometric analysis, all information was gathered, including the number of publications and citations, publication year, titles, authors, affiliations, keywords, countries, journals, and references.

### Analytical tools

In this study, CiteSpace (version 6.1.R3) and VOSviewer (version 1.6.18) were used to perform the bibliometric analysis.

CiteSpace is a bibliometrics and visual analysis tool developed by Professor Chen Chaomei that may be used to investigate key points, internal architecture, and possible trends in certain fields (Chen, 2004). CiteSpace 6.1.R3 was used by our team to investigate and illustrate high-frequency keyword patterns, co-citation references, and citation bursts. CiteSpace is configured as follows: The period span of the selection is from 2004 to 2022 with a 1-year time slice. To simplify the network and emphasize the basic features, the pathfinding pruning method and the minimal spanning tree algorithm are utilized. Set top = 50 as the threshold, and leave the other variables at their defaults in CiteSpace. In Citespace, several network topology parameters were used to describe the structure of complicated networks. The betweenness centrality is a metric for determining the importance of a node in a network. It represents how much a node in the network diagram acts as an “intermediary” for other nodes, and acts as “bridge” in the network (Freeman, 1977; Brandes, 2001). In cluster analysis, two essential evaluation indicators, modularity Q and mean silhouette, were displayed. If Q > 0.3, the clustering structure is significant enough; if the mean silhouette is > 0.5, the clustering results are compelling (Chen et al., 2010). The function of burst detection can detect large changes in the reference count in a certain period of time. It is used to find the decline or rise of a subject word or keyword.

Complex bibliometric networks, such as collaboration and temporal trends across countries were shown using VOSviewer (van Eck and Waltman, 2010). The number of publications is represented by the size of the nodes; the strength of the link is represented by the thickness of the line, and the colors of the nodes signify distinct groups or times. Total link strength means the number of links between this node and other nodes. The higher the total link strength value, the stronger the collaboration between the node and other nodes.

## Results

### Overall distribution

**FIGURE 2:**
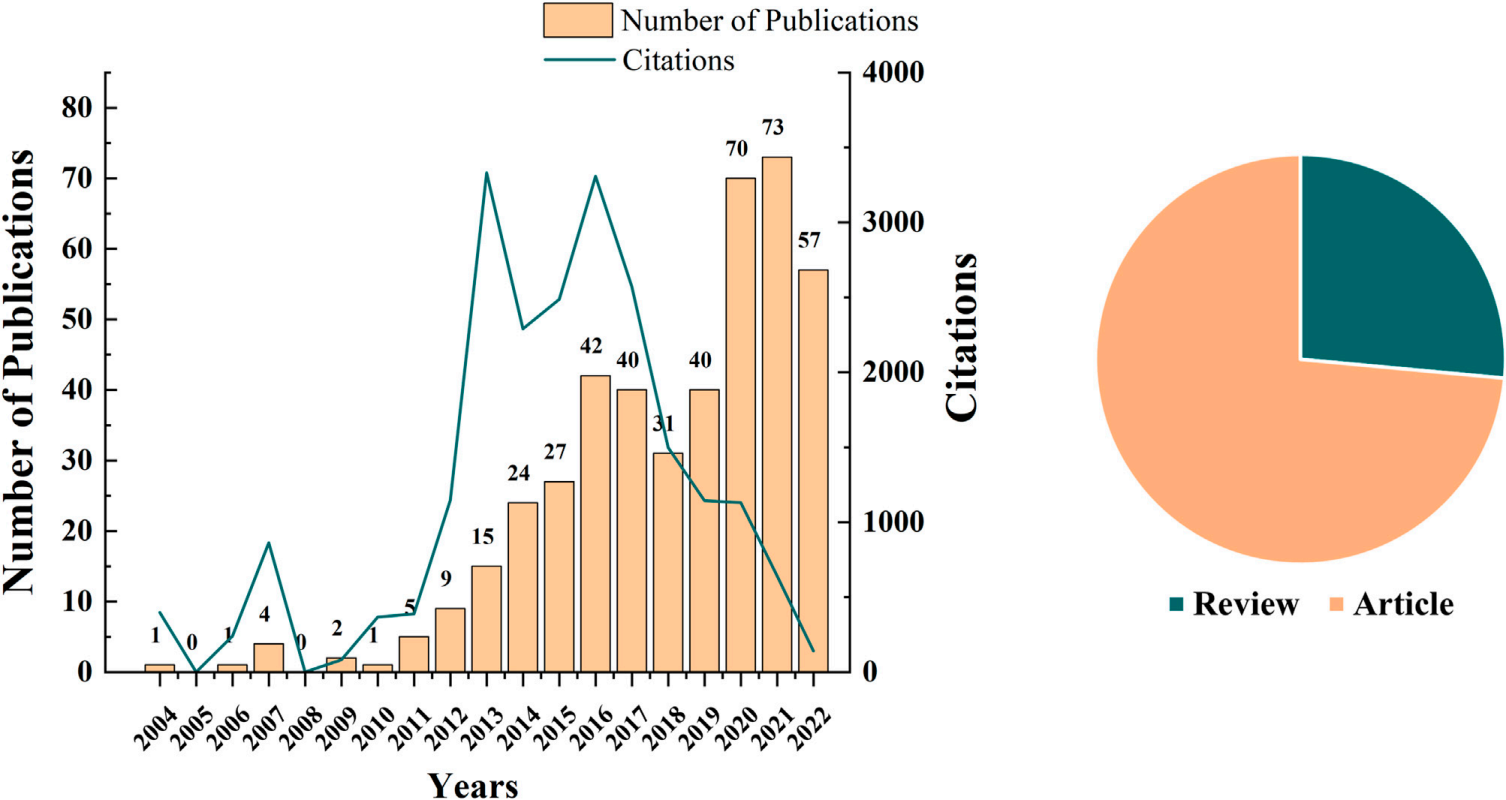
Annual publication volume and type of publications in UPR^mt^.

WoSCC included 442 publications between 2004 and 2022, comprising 325 (73.6%) articles and 117 (26.4%) reviews from 215 journals. As of 30 November 2022, in the WoSCC citation report, the total number of citations of these articles is 22,018, the average number of citations of each paper is 49.81, and the h index reaches 76. The number of annual publications on the universal periodic review (UPR^mt^) has been rising since 2004 and has increased rapidly in 2020 (Figure 2). This shows that UPR^mt^ has become a topic of increasing concern in recent years and has attracted extensive attention from scholars. As shown in Table 1, the top 10 journals accounting for 20.13% of these publications. *International Journal of Molecular Sciences* had the highest number of publications (159 citations), followed by *Scientific Reports* (217 citations), and *Aging Cell* (230 citations). Furthermore, while *Nature* was ranked tenth, its impact factor (69.504) was significantly higher than that of the majority of the journals listed. A closer analysis of the data reveals that there is no clear relationship between the number of publications and the impact factor (IF) of the journals. For example, while *PLOS One* had the highest number of citations (587) among the top 10 journals, its impact factor (3.752) was relatively low compared to other journals on the list. Similarly, *Free Radical Biology and Medicine* had a relatively low number of publications (7) but had a relatively high IF (8.101). It is also worth noting that some journals on the list, such as *Aging Cell and Proceedings of the National Academy of Sciences of the United States of America*, had a high number of citations and a high IF, indicating their significance in the field of UPR^mt^ research.

**TABLE 1:** The top 10 journals related to UPR^mt^.

Rank	Journal	Documents	IF (2021)	Citations	Average citation
1	International Journal of Molecular Sciences	13	6.208	159	12.23
2	Scientific Reports	10	4.997	217	21.70
3	Aging Cell	9	11.005	230	25.56
4	Cell Death & Disease	9	9.696	287	31.89
5	PLoS One	9	3.752	587	65.22
6	Proceedings of the National Academy of Sciences of the United States of America	9	12.779	339	37.67
7	Cell Reports	8	9.995	199	24.88
8	PLOS Genetics	8	6.020	342	42.75
9	Free Radical Biology and Medicine	7	8.101	197	28.14
10	Nature	7	69.504	1,506	215.14

### Countries, institutes and authors

**FIGURE 3:**
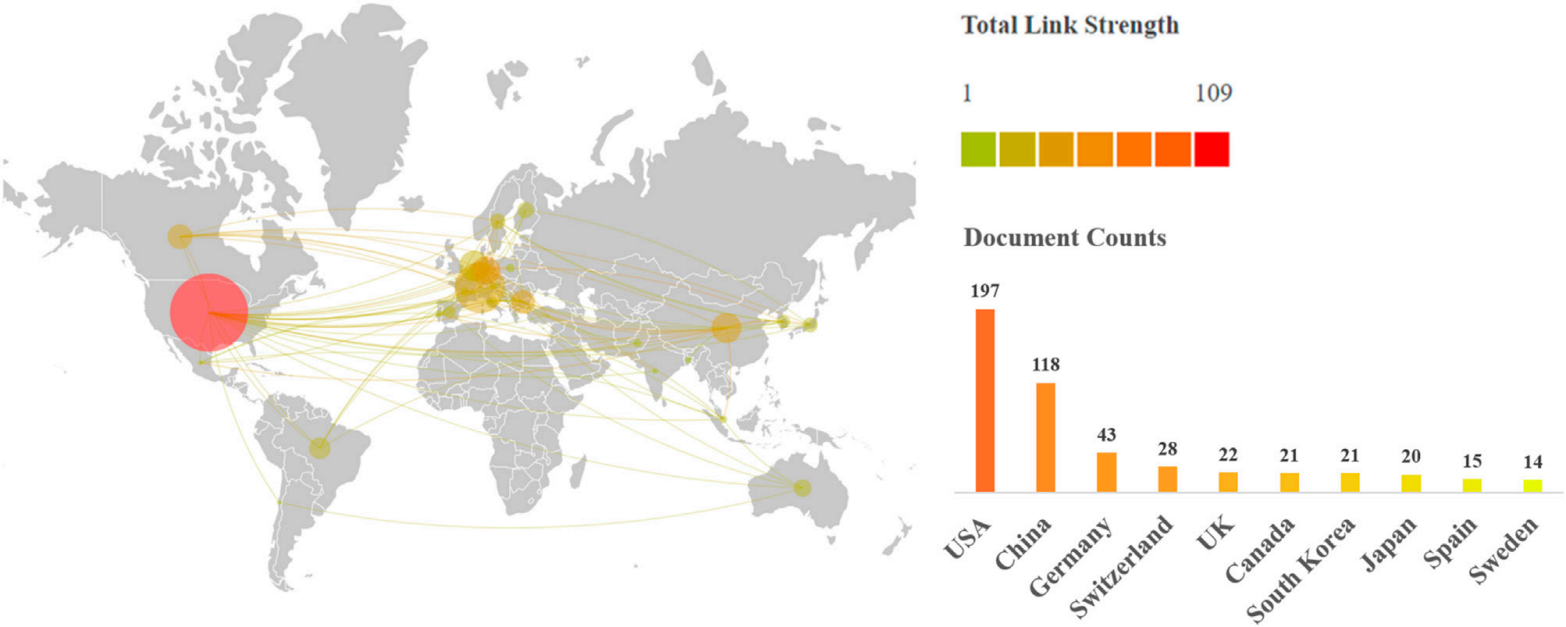
Country collaboration network and top 10 countries in UPR^mt^.

**FIGURE 4:**
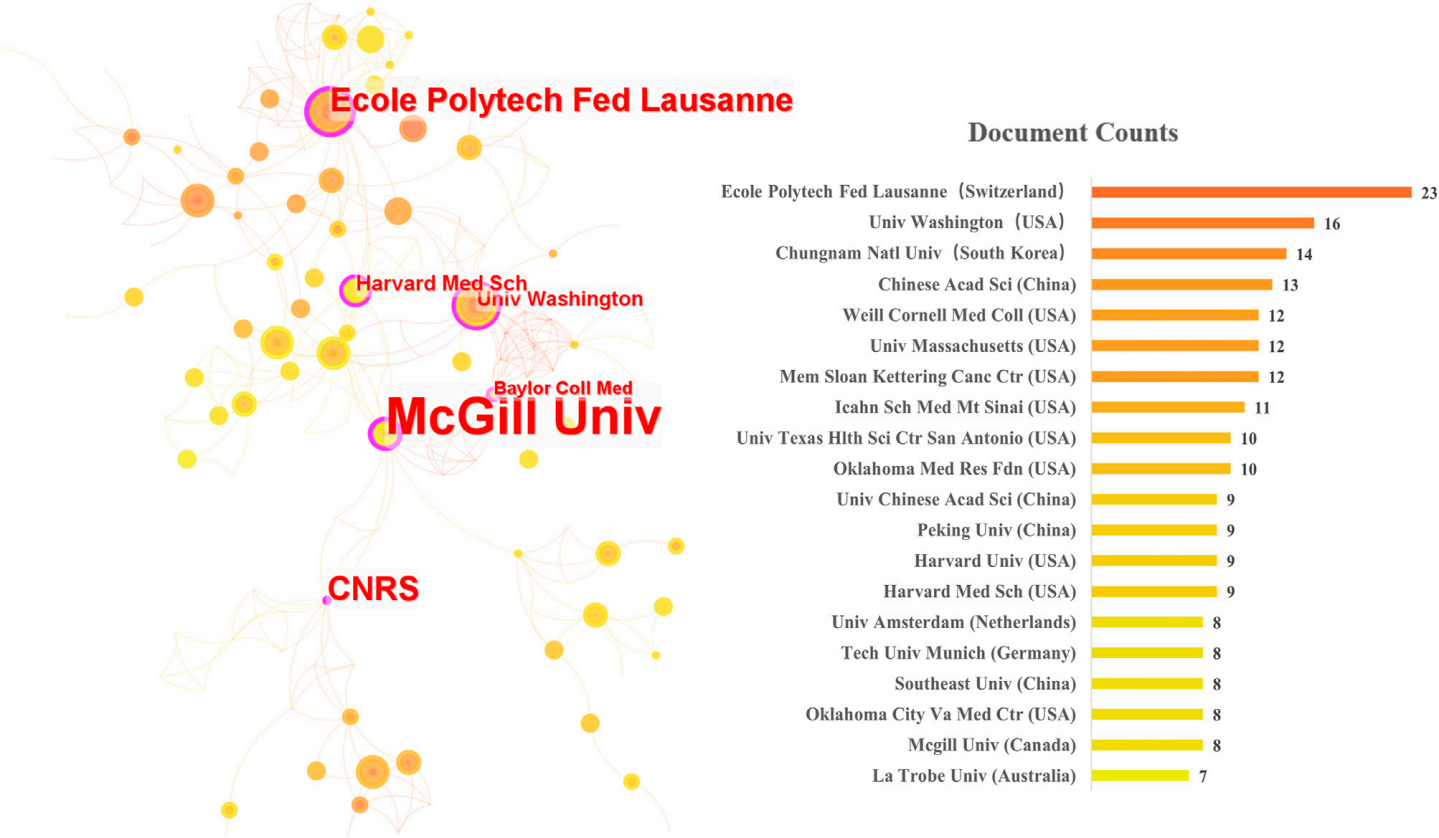
Institution collaboration networkand top 10 affiliations in UPR^mt^.

Between 2004 and 2022, 184 countries, 1,402 institutions, and 2421 authors published UPR^mt^ -related articles/reviews. Figure 3 shows the countries by the number of publications and their collaborations. The United States ranked first with 197 documents, China ranked second with 118, and Germany ranked third with 43. The United States and China account for the biggest proportion of document publications, while China has lower total link strength compared to the United States. This implies that collaboration between China and other nations is limited. The top 20 institutions in document counts and their collaboration network are presented in Figure 4. Ecole Polytech Fed Lausanne from Switzerland published the most papers with 23, followed by the Univ Washington (16) and the Chungnam Natl Univ (14). The visual collaboration network is based on the betweenness centrality value. Notably, the McGill Univ form Canada got the highest betweenness centrality value (the biggest font size of node). As shown in Table 2, Johan Auwerx from Ecole Polytechnique Federale de Lausanne ranked first with 5,337 citations among the top 15 authors. Cole M Haynes from UMass Chan medical school ranked second with 3,777 citations and, at the same time, was the author with the highest number of documents. In additions, Johan Auwerx has the highest average citations per document (242.59), followed by Dongryeol Ryu (414.00) and Norman Moullan (333.17). This suggests that their research has been more impactful and influential in the field of UPR^mt^ compared to other authors on the list.

**TABLE 2:** Top 15 most cited authors in the field of UPR^mt^.

Rank	Author	Affiliations	Documents	Citations	Average citations
1	Johan Auwerx	Ecole Polytech Fed Lausanne (Switzerland)	22	5,337	242.59
2	Cole M Haynes	UMass Chan Med Sch (United States)	24	3,777	157.38
3	Dongryeol Ryu	Sungkyunkwan Univ (South Korea)	6	2484	414.00
4	Norman Moullan	Ecole Polytech Fed Lausanne (Switzerland)	6	1999	333.17
5	Riekelt H Houtkooper	Univ Amsterdam (Netherlands)	7	1829	261.29
6	Mark W Pellegrino	Univ Texas (United States)	11	1,370	124.55
7	Xu Wang	Ecole Polytech Fed Lausanne (Switzerland)	6	985	164.17
8	Eduardo R Ropelle	Univ Estadual Campinas (Brazil)	6	947	157.83
9	Virginija Jovaisaite	Ecole Polytech Fed Lausanne (Switzerland)	5	562	112.40
10	Doris Germain	Icahn Sch Med Mt Sinai (United States)	12	543	45.25
11	Hyon-Seung Yi	Chungnam Natl Univ (South Korea)	8	331	41.38
12	Minho Shong	Chungnam Natl Univ (South Korea)	8	318	39.75
13	Joon Young Chang	Chungnam Natl Univ (South Korea)	6	304	50.67
14	Shauna Hill	Univ Texas (United States)	7	238	34.00
15	Saet-Byel Jung	Chungnam Natl Univ (South Korea)	5	221	44.20

### Keywords

**FIGURE 5:**
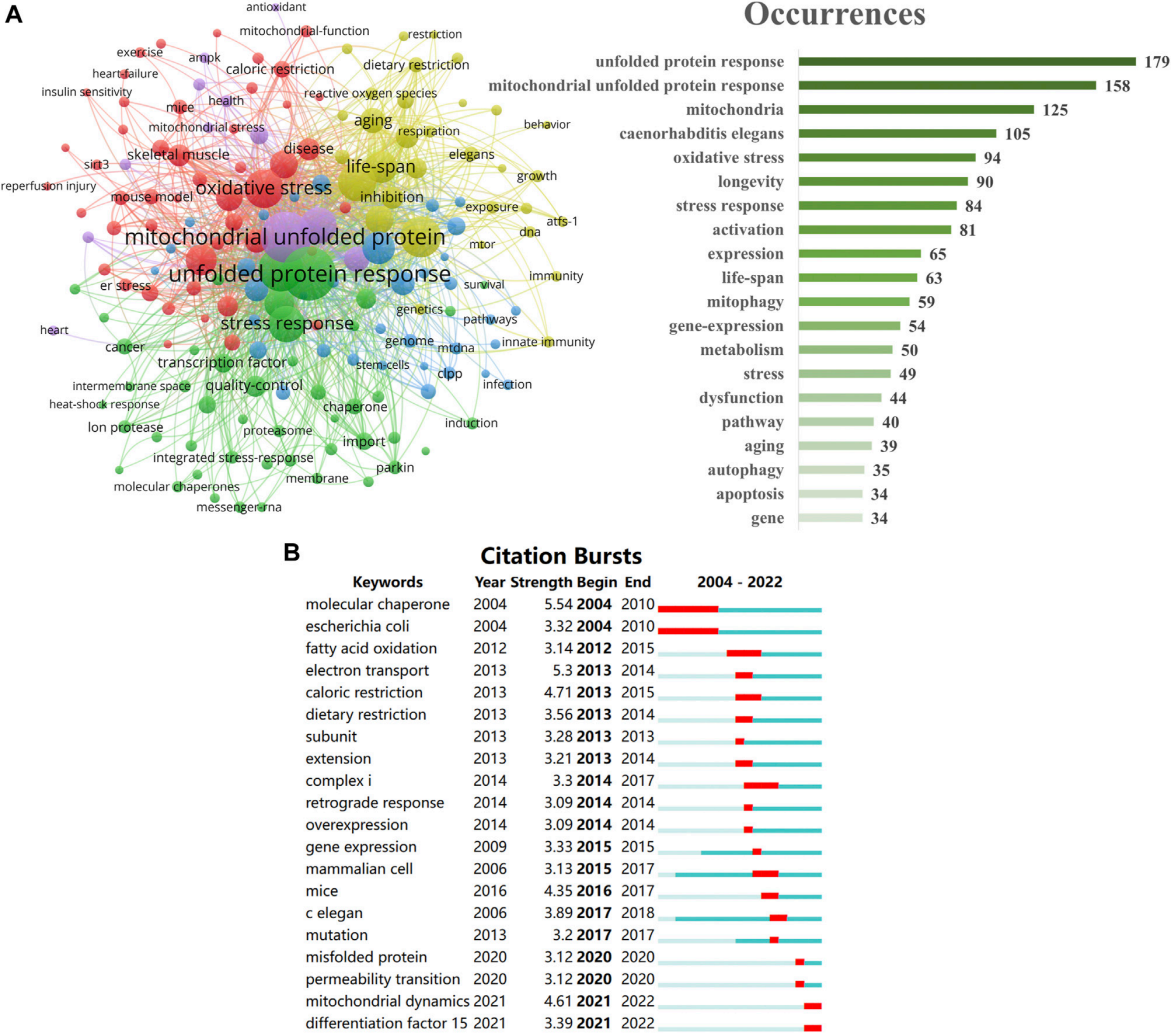
Keyword co-occurrence **(A)** and top 25 burst **(B)** in the field of UPR^mt^.

Keyword analysis can be used to investigate research hotspots and frontiers in a field. A total of 841 author keywords were identified. The keyword co-occurrence (**A**), keyword burst (**B**), and the top 25 keywords are presented in Figure 5. In Figure 5A, each keyword is represented by a node, and the thickness of the lines connecting the nodes indicates the strength of the co-occurrence relationship between them. Nodes that are closer to each other are more closely related in terms of their co-occurrence patterns. The maps can help researchers identify important topics or research areas by visualizing the patterns of keyword co-occurrence. These keywords were divided into 5 clusters (exhibited in 5 colors: red, yellow, blue, green and purple) by VOSviewer, including 173 nodes, 4,548 links, and a total link strength of 10,610. Occurrences is listed on the right side of Figure 5A. In addition to keywords with the same literal meaning as the subject, the following keywords with the highest occurrences and biggest node are “*caenorhabditis elegans,*” “oxidative stress,” “longevity,” with 105, 94 and 90 occurrences. The intensity of the citation bursts is a good indicator of the study’s hotspots and rising frontiers over time (Pei et al., 2022). The strongest and earliest citation burst was “molecular chaperone,” with a score of 5.54. And the latest one was “differentiation factor 15,” which means an emerging research direction (Figure 5B).

### References and Co-Cited references

The top 10 documents with highest citations included 3 reviews and 7 research articles (Table 3). The article with the highest citations was authored by Amrita M Nargund *et al.* (cited 1,122 times). Amrita M Nargund *et al.* discovered that cells monitor mitochondrial import efficiency *via* ATFS-1 (activating transcription factor associated with stress-1) to coordinate the level of mitochondrial dysfunction with the protective transcriptional response (Nargund et al., 2012a). Co-cited reference refers to the relationship between two publications when a third document references them simultaneously. The higher the co-cited reference frequency of these two publications, the closer their academic connection and “distance.” Citespace uses the statistical method of cluster analysis to classify the publications in the discipline field based on this “distance” and uses the graphical representation to identify and evaluate the subdivision of the discipline field visually. Through Citespace, we built a visual network of co-cited references, and generated a timeline view. A total of 12 clusters were extracted by cluster analysis (the skipped number means that this cluster contains less than 10 documents). The modularity Q was 0.7022, and the mean silhouette value was 0.8398. According to the cluster analysis, the most highly regarded cluster with the most nodes and references is “uprmt regulation”, followed by “sh-sy5y cell” and “protecting organelle protein homeostasis”. The “protein” is an early field of UPR^mt^ and “sh-sy5y cell " and “*C. elegans* " are the current hot topics.

**TABLE 3:** Top 10 highest citation publications in the field of UPR^mt^.

Rank	First author	Journal	Year	2021 IF	Citations
1	Amrita M Nargund	Science	2012	63.832	199
2	Riekelt H Houtkooper	Nature	2013	69.504	164
3	Cole M Haynes	Developmental Cell	2007	13.417	150
4	Takunari Yoneda	Journal of Cell Science	2004	5.235	142
5	Cristina Benedetti	Genetics	2006	4.402	105
6	Laurent Mouchiroud	Cell	2013	66.850	97
7	Cole M Haynes	Journal of Cell Science	2010	5.235	77
8	Mark W Pellegrino	Nature	2014	69.504	75
9	Mark W Pellegrino	Biochimica et Biophysica Acta-molecular Cell Research	2013	5.011	67
10	Virginija Jovaisaite	Journal of Experimental Biology	2014	3.308	67

## Discussion

Multiple software packages were employed in this work to conduct a bibliometric analysis of global scientific outputs relevant to UPR^mt^ published between 2004 and 2022. The WoSCC records were evaluated from several angles, with the results shown in tables and knowledge network maps. The recent development track can be divided into three stages: 2004–2010 that exhibited a slow development; 2011–2016 that exhibited a stable development; 2019–2021 that exhibited a rapid development. These studies suggest that UPR^mt^ is a key link in maintaining mitochondrial protein homeostasis, and the importance of UPR^mt^ to mitochondrial function is increasingly garnering the attention of more researchers.

The United States was the most productive country that promotes the research of UPR^mt^, and 10 of the top 20 productive institutions are from there. The Univ Washington (published 16 articles, cited 816 times) was the main representative. Johan Auwerx from Ecole Polytech Fed Lausanne got most of the citations (published 22 articles, cited 5,337 times). He pioneered the use of systematic, cross-species genetics and multi-layer “omics” gene discovery methods to map complex signal networks that control metabolism. His team discovered that the NAD + - neurons regulating axis is closely linked with health and life span, which is a significant advance in anti-aging research (Mouchiroud et al., 2013; Cantó et al., 2015). In the top 10 highest citation publications, scholars have focused on the related pathway mechanism of UPR^mt^ (Benedetti et al., 2006; Haynes et al., 2007; Pellegrino et al., 2014; Cantó et al., 2015) and further explored how it can be applied to aging related diseases, such as cancer, metabolic diseases, and neurodegenerative diseases (Jovaisaite et al., 2014).

The analysis of keywords provides valuable insights into the research topic centered around UPR^mt^. The frequent occurrence of “*caenorhabditis elegans*” suggests that this model organism is a crucial tool in studying UPR^mt^. *Caenorhabditis elegans*, a free-living nematode, is commonly used in life sciences research, particularly in the study of aging, owing to its simplicity and short lifespan. The high frequency of “oxidative stress” suggests that the research topic involves the cellular response to oxidative stress, a major factor in several diseases, including cancer, cardiovascular disease, and neurodegenerative diseases. UPR^mt^ plays a significant role in the cellular response to oxidative stress (Zhao, 2019), regulating protein folding and quality control in mitochondria. By studying UPR^mt^ in the context of oxidative stress, researchers can uncover the mechanisms underlying these diseases and identify potential therapeutic targets. The high frequency of “longevity” indicates that the research topic involves the study of lifespan and aging, closely related to the role of UPR^mt^ in the cellular aging process. UPR^mt^ regulates protein quality control and mitochondrial function, both key factors in aging. Therefore, studying UPR^mt^ in the context of lifespan and aging can shed light on the mechanisms underlying these processes and identify potential interventions to extend healthy lifespan. Additionally, other high-frequency keywords, such as “mitochondria,” “stress response,” “activation,” “expression,” “life-span,” and “mitophagy,” offer further insights into the research topic.

The burst of “molecular chaperone” signifies the significance and prominence of this keyword in the early stages of UPR^mt^ research. Molecular chaperones are a crucial group of proteins that aid in protein folding and quality control, and their relevance to the UPR and UPR^mt^ pathways cannot be overlooked. The increase in the use of “molecular chaperone” implies that early research on UPR^mt^ was focused on comprehending the role of molecular chaperones in protein folding and quality control within the context of mitochondrial stress. The burst “differentiation factor 15″suggests an upsurge in interest in the role of this protein in UPR^mt^. Growth differentiation factor 15 (GDF15) is a stress-responsive cytokine that is involved in various physiological processes, including inflammation, metabolism, and tissue repair. Recent studies have proposed that GDF15 may play a crucial role in UPR^mt^ by serving as a mitochondrial stress signal, activating the UPR^mt^ pathway and enhancing mitochondrial protein quality control (Johann et al., 2021). The increased focus on GDF15 in recent years is likely a reflection of the growing recognition of its potential as a therapeutic target for diseases involving mitochondrial dysfunction, such as neurodegenerative and metabolic disorders.

### Research hotspots and trends

**FIGURE 6:**
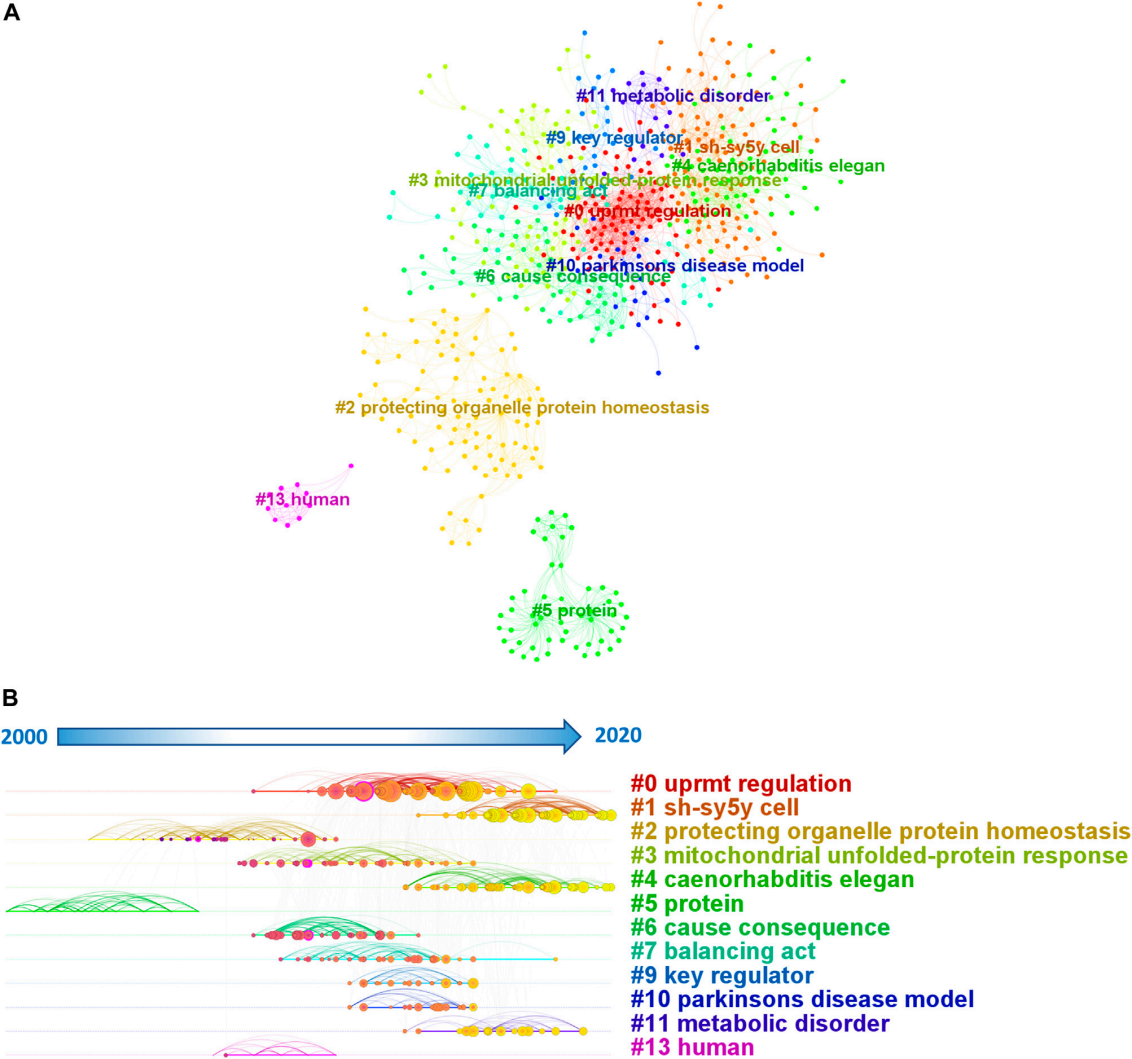
Co-citation network **(A)** and timeline view **(B)** in UPR^mt^.

By analyzing the co-citation reference timeline view, the past research hotspots and the current research trends could be found. The timeline view show in Figure 6 illustrates the following findings: early research concentrated on “#5 protein,” interim studies concentrated on “#2 protecting organelle protein homeostasis,” “#6 cause consequence,””#0 uprmt regulation,” whereas current studies concentrated on “#1 sh-sy5y cell” and “#11 metabolic disorder”.

### Initial period

Chaperones were initially thought to play important roles in nascent polypeptide folding, assembly of multimeric protein complexes, protein translocation across membrane barriers, integration into membranes, and protein degradation (Bukau and Horwich, 1998; Horwich et al., 1999; Hartl and Hayer-Hartl, 2002). Cluster #5“protein” occurred during this period. It is not surprising to see this result as proteins are the fundamental building blocks of cells and their functions. Proteins are also essential for the proper functioning of mitochondria, which are the main organelles affected by UPR^mt^. A 2002 study discovered that a non-folding mitochondrial matrix protein activates genes encoding mitochondrial chaperones in mammalian cells, directly confirmed the existence of UPR^mt^ (Zhao et al., 2002). During the initial period, researchers were primarily focused on identifying the role of proteins in UPR^mt^. This period was critical for understanding the mechanisms by which UPR^mt^ works and the specific proteins involved. The researchers during the Initial period were trying to identify the proteins that could activate UPR^mt^ and the mechanisms by which UPR^mt^ could regulate protein homeostasis in mitochondria. These findings laid the foundation for the Development and Recent periods of research on UPR^mt^.

### Development period

The cluster #2 protecting organelle protein homeostasis directly summarized the period investigators’ understanding about the UPR^mt^. The investigation of the UPR^mt^ in *C. elegans* had resulted in a deeper understanding of this stress response pathway (Haynes et al., 2007; Haynes et al., 2010). The existence of the UPR^mt^ enables for variations in chaperone protein expression that are closely connected with the degradation of a fraction of accumulated unfolded proteins, ensuring protein folding homeostasis at the levels of protein folding and removal. Also in *C. elegans*, UPR^mt^ was found to affect lifespan with both extension and reduction (Ventura and Rea, 2007; Artal-Sanz and Tavernarakis, 2009). Given the central importance of mitochondria in aging and age-related diseases, this has facilitated studies on the impact of the UPR^mt^ on human disease. The cluster #0 uprmt regulationand is the highest regarded cluster, implying that the exploration of regulatory mechanisms of the UPR^mt^ was the focus of this period. In contrast to *C. elegans*, the regulation of the UPR^mt^ in mammals is potentially more complex (Owusu-Ansah et al., 2013; Pirinen et al., 2014; Fiorese et al., 2016). Through experiments on mammalian, the UPRmt had been discovered with novel and unexpected roles that went well beyond its originally identified role in restoring mitochondrial proteostasis. In addition to being closely related to aging, UPR^mt^ had also been found to be closely related to a variety of diseases, such as Alzheimer’s disease (Beck et al., 2016; Sorrentino et al., 2017), Parkinson’s disease (Martinez et al., 2017) and cancers (Deng and Haynes, 2017).The cluster #10, “Parkinson’s disease model,” during the development phase also suggests a growing interest in understanding the role that UPR^mt^ may play in the pathogenesis of neurodegenerative disorders such as Parkinson’s disease (Hu et al., 2021). By studying UPR^mt^ in the context of these diseases, researchers may identify new targets for therapeutic intervention and develop new treatments for these debilitating conditions. Cluster #13, “human,” during the development phase also indicates an important shift in the focus of UPR^mt^ research. While previous studies may have primarily focused on animal models or cell lines, the rising interest in studying UPR^mt^ in human cells and tissues reflects a recognition of the significance of understanding this process in the context of human health and disease. This shift towards human-based research is likely to yield critical insights into the underlying mechanisms of UPR^mt^ and the ways in which it can be targeted for therapeutic purposes. Cluster #6, “cause consequence,” is noteworthy as it suggests a growing interest in comprehending the causal relationships between UPR^mt^ and other cellular processes or disease states. Cluster #9, “key regulator,” is also noteworthy, indicating a growing interest in identifying the key molecular players involved in UPR^mt^ regulation and function. By identifying these key regulators, researchers may develop more targeted and effective therapies for diseases that involve mitochondrial dysfunction. Other clusters that emerged during the development phase include cluster #3, “mitochondrial unfolded-protein response,” which reflects an increasing understanding of the role that UPR^mt^ plays in maintaining mitochondrial protein quality control. Clusters #7, “balancing act,” suggest a growing recognition of the intricate and dynamic nature of UPR^mt^ regulation and the need to balance this process with other cellular functions.

### Recent period

As mentioned above, neurodegenerative diseases had become a current research hotspot. Sh-sy5y is one of the most commonly used cell lines in brain science research. Mitochondrial dysfunction occurs has a significant impact on neurodegenerative diseases. A substantial buildup of unfolded, misfolded, or invalid proteins is a common symptom of neurodegenerative diseases. It has been discovered that UPR^mt^ is activated by the mevalonic acid route and the ceramide pathway, preventing amyloid–β (Aβ) aggregation and alleviating AD symptoms (Shen et al., 2019); Pink-1 activates ATFS-1-dependent UPR^mt^, which increases dopaminergic neuron survival and thereby alleviates Parkinson’s disease (Cooper et al., 2017); UPR^mt^ suppresses the production of polyQ aggregates, which may help to prevent Huntington’s disease (Fu et al., 2019). Besides, cluster #11 (metabolic disorder) similarly represents a recent research hotspot. In recent years, the link between UPR^mt^ and numerous metabolic diseases has been discovered. For example, GDF15 as a serum biomarker predicts liver diseases including Non-alcoholic fatty liver disease and advanced liver fibrosis in humans. Its promoter was found to be directly bound by C/EBP-homologous protein (CHOP is part of the unfolded protein response signaling pathway in the mitochondria and endoplasmic reticulum) and activate its transcription. The cluster #4“*Caenorhabditis elegans*” is highly relevant in the recent period. *C. elegans* has traditionally been used to study fundamental aspects of key biological processes such as apoptosis, aging, and gene expression regulation. With the advent of large-scale screening platforms, this invertebrate has also become an important tool in the drug discovery industry for identifying new drugs and drug targets. High-throughput screening of *C. elegans* has indeed helped to break through a variety of candidate compounds involving broad areas, including neurodegeneration, pathogen infection, and metabolic disorders (Giunti et al., 2021).

Through our analysis of recent research, we have found that the UPR^mt^ plays a crucial role in neurodegenerative diseases, cancer, and liver diseases. However, further studies are needed to explore the regulatory mechanisms of UPR^mt^ in diseases. The promising potential of UPR^mt^ research in human disorders offers fresh insights into therapy for various diseases. The advancement of UPR^mt^ research may impact human life by presenting new potential therapeutic targets. This manuscript offers valuable insights into the frontiers and trends of UPR^mt^ research, providing clinicians and scientists with information on potential collaborators and cooperative countries. Additionally, it highlights possible topics or groups that could contribute to advancements in this field of research. By exploring these areas of interest, researchers can identify new perspectives that could ultimately lead to improved clinical applications, benefiting patients and advancing the field of UPR^mt^.

## Limitation

Although bibliometric analysis gives more insight into research topics and trends than traditional assessments, it has several limitations. For starters, this article excludes non-English literature, which might be a source of prejudice. Furthermore, because of the trustworthiness of the publications and citations, the data utilized in this work was acquired entirely from the WoSCC database. We are unable to undertake relevant analysis (such as co-citation analysis) on PubMed or other databases (lack of information on references) due to software limitations, which enhances the study bias to a certain extent. The local database, for this reason, has fewer literature and journals than other databases, resulting in less comprehensive study findings. Furthermore, we examined the characteristics of the data we collected to highlight the most essential aspects. As a result, certain information may be missed.

Furthermore, the majority of this study’s results are based on a machine algorithm, which is severely lacking in artificial induction. Finally, due to the sensitivity of machine algorithms, several new research fields of UPR^mt^ may not have been included.
